# Antioxidant Responses and Redox Regulation Within Plant-Beneficial Microbe Interaction

**DOI:** 10.3390/antiox13121553

**Published:** 2024-12-18

**Authors:** María-Cruz González, Thomas Roitsch, Chandana Pandey

**Affiliations:** 1 Instituto de Bioquímica Vegetal y Fotosíntesis, Universidad de Sevilla and Consejo Superior de Investigaciones Científicas, 41092 Sevilla, Spain; 2 Departamento de Bioquímica Vegetal y Biología Molecular, Facultad de Biología, Universidad de Sevilla, 41012 Sevilla, Spain; 3 Copenhagen Plant Science Centre, Faculty of Science, University of Copenhagen, 1870 Frederiksberg, Denmark; roitsch@plen.ku.dk; 4 CzechGlobe—Global Change Research Institute CAS, 603 00 Brno, Czech Republic

**Keywords:** biopriming, biocontrol, redox regulation, antioxidant responses, biotic stress, disease defense, beneficial microbe, symbiosis, sustainable agriculture

## Abstract

The increase in extreme climate events associated with global warming is a great menace to crop productivity nowadays. In addition to abiotic stresses, warmer conditions favor the spread of infectious diseases affecting plant performance. Within this context, beneficial microbes constitute a sustainable alternative for the mitigation of the effects of climate change on plant growth and productivity. Used as biostimulants to improve plant growth, they also increase plant resistance to abiotic and biotic stresses through the generation of a primed status in the plant, leading to a better and faster response to stress. In this review, we have focused on the importance of a balanced redox status for the adequate performance of the plant and revisited the different antioxidant mechanisms supporting the biocontrol effect of beneficial microbes through the adjustment of the levels of reactive oxygen species (ROS). In addition, the different tools for the analysis of antioxidant responses and redox regulation have been evaluated. The importance of redox regulation in the activation of the immune responses through different mechanisms, such as transcriptional regulation, retrograde signaling, and post-translational modification of proteins, emerges as an important research goal for understanding the biocontrol activity of the beneficial microbes.

## 1. Introduction

Human activity, and specifically the resulting increase in CO_2_ concentration in the atmosphere, has led to an increase in global temperature, known as global warming. This in turn is responsible for climate change, including waves of high temperatures, drought, soil salinity, flooding, and chilling. All these conditions impose stress on plant growth that results in a decrease of crop yield, threatening the nourishment of an ever-increasing population. As a consequence, the decrease in the number of undernourished people observed from the 1990s on, due to UN policies [1], was slowed down from 2007 due, among other factors, to the impact of climate change on agriculture. Moreover, from 2014–2018 the number stopped decreasing [2], this being attributed to the higher tendency of extreme climate events.

Plants possess different mechanisms to cope with stress situations [3]. Drought and high temperature affect membrane and protein stability and generate an increase in the production of reactive oxygen species (ROS), ultimately leading to an unbalanced metabolism and reduced photosynthetic activity [4]. To ensure survival under drought and heat stress, plants increase the synthesis of compatible solutes, that prevent the loss of water and play osmoprotective roles, maintaining membrane integrity and protecting proteins from denaturation. In addition, heat stress tolerance is dependent on the production of an increased amount of heat shock proteins (HSPs), that maintain protein homeostasis. In both cases, an adjustment of ROS levels is required to avoid damage to proteins, lipids, nucleic acids, and chlorophyll. Biotic stress associated with infection by pathogens, addressed in depth in this manuscript, is also associated with an increase in the production of ROS, a key event for the activation of plant immune responses. Moreover, ROS could alter the activity of redox-regulated enzymes. Therefore, an increase in the different antioxidant systems of the plant, reducing ROS levels, is key for plant survival under adverse conditions [5].

Different simulation models suggest that in the coming years productivity of the main crops (wheat, rice, maize, and soybean) will be decreased, due to an increasing degree of climate change [1,6,7,8]. In addition, the chemical composition of the harvested products could be altered, leading to a decrease in nutritional quality [9]. Therefore, efforts must be undertaken to generate crops that can better resist the actual adverse conditions. With this goal, traditional breeding and different genetic approaches have been used to analyze the effect of particular abiotic stresses on plant growth and productivity. However, the effect of the combination of stresses on plant responses has been overlooked for a long time [6]. In fact, climate change conditions subject plants to simultaneous abiotic stresses, provoking increased damage to the plants. In addition, warmer climates are responsible for the spread of emerging infectious diseases, related to the appearance of new pathogenic strains [6,10]. Since plants are subjected to multiple simultaneous stresses, understanding and improving plant tolerance to stress combinations is a must nowadays.

Plants are associated with a variable number and diversity of microbes. They are referred to as microbiota and collectively encode the microbiome of a plant. The microbes can live in a neutral, beneficial, or pathogenic interaction [11,12]. The beneficial effects of the microbiota can be divided into two functions [12]: (1) Improvement of environmental adaptability, ecological plasticity, and fitness and (2) support of plant and root growth, development, and architecture. These functions are notably relevant for adaptation within climate change scenarios and also for sustainable and environmentally friendly agricultural production by their application as biostimulants to sustainably improve stress resilience and secure yield and yield stability [13]. Beneficial microorganisms not only upregulate plant growth under control conditions but also improve plant stress tolerance [14], inducing a primed state in the plant that results in a quicker and more efficient response to the stress conditions [15], including the rapid activation of non-enzymatic and enzymatic ROS detoxification mechanisms. In this manuscript, we will review the current knowledge on antioxidant responses elicited by plant-beneficial microbe interaction and discuss the effect on redox regulation, key for the activity of many photosynthetic enzymes and transcription factors. The knowledge on the effect of microbes on plant resistance towards biotic stress will also be revised.

## 2. Antioxidant Responses and Redox Regulation

Increased ROS production is a common element in plant response to abiotic and biotic stresses. These molecules (e.g., H_2_O_2_, ^1^O_2_, and O_2_^−^), with a higher reactivity than O_2_, are by-products of aerobic metabolism that play a key role as signaling molecules in plant responses to adverse conditions. In addition, ROS are also involved in the regulation of multiple processes of plant development [16]. However, since high ROS levels may damage the cell, inhibit plant growth, and eventually result in cell death, ROS levels must be finely tuned through a balance between production and scavenging mechanisms [16,17]. Stress conditions can alter this balance and plants with a higher tolerance to abiotic and biotic stresses due to priming with beneficial microorganisms show an increase in ROS detoxification mechanisms to prevent the effects of ROS accumulation. As an example, an oxidative burst is characteristic of plant defense against pathogens, due to the activity of plasma membrane NADPH oxidases, which generate O_2_^−^. In addition, apoplastic peroxidases contribute to H_2_O_2_ release, which is responsible for the activation of plant defense responses [18]. Hypersensitive response, preventing the spread of biotrophic pathogens in the plant, is also associated with an oxidative burst at the site of infection. Under abiotic stress, ROS production can be due an altered metabolism (metabolic ROS) but also a result of stress perception and activation of plasma membrane NADPH oxidases (signaling ROS), generating a ROS signature characteristic of each cell [19]. Metabolic ROS are associated with the generation of oxygen in the photosynthetic electron transport chain and its consumption by the photosystems within the chloroplasts, the main contributors to ROS production.

**Figure 1:**
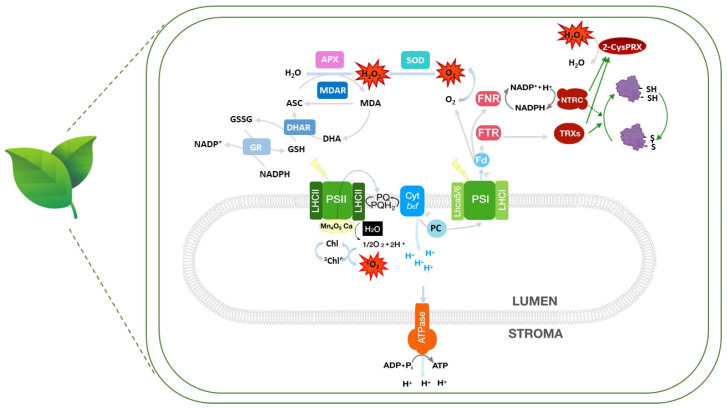
Reactive oxygen species (ROS) production, ROS enzymatic detoxification, and redox regulation within the chloroplast. In the chloroplast, ROS are produced due to energy transference at PSII (singlet oxygen, ^1^O_2_) and electron transference to oxygen in PSI (superoxide anion, O_2_^−^). Superoxide anion is dismutated to hydrogen peroxide, which is in turn reduced by APX and ASC/GSH, through the ASC-GSH cycle. APX uses ascorbate to reduce hydrogen peroxide, generating monodehydroascorbate (MDA), which can be in turn reduced by monodehydroascorbate reductase (MDAR) using reducing equivalents. Spontaneous dismutation of MDA generates dehydroascorbate (DHA). Regeneration of ASC is catalyzed by dehydroascorbate reductase (DHAR) using GSH as source of reducing equivalents. GSH reduction is driven by glutathione reductase (GR). Redox regulation (represented by green arrows) in the chloroplast is dependent on disulfide–dithiol exchange of target proteins by Trxs, which are in turn reduced by FTR using Fd as source of reducing power. NTRC uses NADPH generated by FNR for the reduction of 2-Cys PRX, leading to hydrogen peroxide reduction. Whereas Trxs can also reduce 2-Cys-PRX, although less efficiently than NTRC, direct involvement of NTRC on protein reduction remains to be determined. Dashed lines indicate not yet established direct interaction. Protein complexes were schematized for a better understanding. Leaves image: https://www.flaticon.com/free-icon/leaves_1635265?term=leaves&page=1&position=4&origin=search&related_id=1635265 (accessed on 16 September 2024).

Since ROS are generated in different cell compartments, different mechanisms operate in each of them to prevent their accumulation [16]. In the chloroplast, non-enzymatic antioxidants, such as carotenoids, tocopherols, and membrane lipids are involved in the detoxification of singlet oxygen (^1^O_2_), produced by energy transference of PSII to O_2_. On the other hand, superoxide anion (O_2_^−^), generated by the transference of one electron to molecular oxygen at PSI, is dismutated into hydrogen peroxide (H_2_O_2_) by superoxide dismutases (SODs) (Figure 1). H_2_O_2_ is also produced in the peroxisome due to the oxidation of 2-phosphoglycolate, generated by the higher oxygenase activity of Rubisco due to the increase in temperature. In the mitochondria, superoxide anion in the matrix, associated with the electron transport chain, is dismutated by SODs. H_2_O_2_ is detoxified by ascorbate (ASC) and ascorbate peroxidases (APXs), glutathione (GSH) and glutathione peroxidases (GPXs), and peroxiredoxins (PRXs) in the stroma (Figure 1), by catalases (CATs) and APXs in the peroxisome, and by APXs, PRXs, and GPXs in the mitochondrial matrix.

The production and detoxification of H_2_O_2_ in the chloroplasts (Figure 1) through 2-Cys PRXs, which are reduced using reduced ferredoxin (Fd_red_) produced by the photosynthetic electron transport chain, through the activity of Fd-tiorredoxin-reductase (FTR) and thioredoxins (Trxs), the so-called Fd/FTR/Trx system, are closely linked to redox regulation of protein activity through dithiol–disulfide exchange. However, although plastidial Trxs can reduce 2-Cys PRXs with different efficiencies, the main reductant in vivo is NTRC, a bimodal enzyme with NADPH-thioredoxin reductase (NTR) and Trx domains in one polypeptide, which can use NADPH as source of reducing power [20]. Since NTRC and Trxs are responsible for the reductive activation of plastidial enzymes, through dithiol–disulfide exchange, the drainage of electrons from Trxs to 2-Cys PRXs in an *ntrc* mutant affects the activation of Calvin–Benson cycle (CBC) enzymes, ATP, and chlorophyll biosynthesis, between other processes, resulting in a decrease in plant growth [21]. In addition, Cys residues are sensitive to oxidation by H_2_O_2_, leading to the formation of sulfenic acid, which can react with glutathione or other thiol groups, in a process reversed by glutarredoxins (Grxs) and Trxs. Otherwise, they can be further oxidized to sulfinic and, irreversibly, to sulfonic acid, provoking protein damage. The de-protonated form of the Cys residue can also react with reactive nitrogen species (RNS), leading to the formation of nitrosothiols, through S-nitrosylation. Some of these post-translational modifications associated with retrograde signaling, gene transcription regulation [22], and plant immune responses [23] will be discussed later.

Trxs, Trx-like proteins, and Grxs are present in different cell compartments as a multigenic family, allowing the adjustment of plant physiology to the environmental conditions, through reduction and oxidation of proteins [24,25]. In the chloroplast, *f*1-2 and *m*1-4 Trxs are involved in redox regulation of carbon fixation and reducing power exchange with the cytosol [26,27] whereas *x* and *y*1-2 Trxs function mainly as antioxidants [28,29], due to their ability to reduce 2-Cys PRXs and PRX-Q, respectively. This is also the case of NTRC, the most efficient reductant of 2-Cys PRXs. Though several interactors of NTRC have been identified, the participation of NTRC in direct redox regulation of protein targets is under discussion (Figure 1) [20,30]. Finally, Trx *z* is a component of plastid-encoded RNA polymerase, essential for chloroplast biogenesis. The mitochondria possess two *o* type Trxs, with Trx *o*1 regulating the Krebs cycle, and a Trx *h*2 able to regulate alternative oxidase (AOX). Finally, *h* Trxs, modulating the response to biotic and abiotic stresses, are widely distributed in the cytosol, nucleus, apoplast, and endoplasmic reticulum. Grxs, using GSH as electron donor, are in turn present in cytosol, plastid, nucleus, and mitochondria. In addition to regulating protein activity, Trxs participate in the regulation of signaling pathways and as chaperones. Furthermore, recent studies have revealed a key role of some Trx-like proteins, such as TrxL2 and ACHT, and even a typical Trx, Trx *f*1, in the oxidation and inactivation of proteins through the transference of electrons to 2-Cys PRX [31].

The study of ROS in plant responses to abiotic and biotic stresses requires the use of various advanced techniques and platforms. These methods enable the precise detection, quantification, and understanding of ROS dynamics, contributing to the validation and extension of existing research. Biochemical assays, especially enzyme activity assays, are pivotal in providing direct insights into the physiological status of plants by measuring the catalytic activities of enzymes involved in ROS detoxification [32,33,34,35]. The traditional assays involve preparing protein extracts from plant materials and subjecting them to spectrophotometric measurements to determine enzyme activities, offering robust and reliable data critical for understanding cell physiological processes [34,35]. Despite being labor-intensive, recent technological advancements have significantly enhanced the throughput of these assays. Robot-based platforms now automate enzyme activity measurements, utilizing a microtiter plate format for end-point determinations and product detection through cycling reactions, increasing throughput by up to 100-fold. Cost-efficient alternatives have also been developed, using a 96-well microtiter plate format and microplate spectrophotometers for real-time monitoring of reaction linearity through kinetic assays [34,35]. These setups enable semi-high-throughput enzyme activity analysis, making the technique accessible to a broader range of researchers, allowing a more complete picture on how abiotic or biotic stresses are affecting enzymatic antioxidant activity. Notably, an analytical platform has been established to assess the activity signature of nine antioxidant enzymes [35]. However, in some cases, a more detailed analysis of the levels and activity of ROS non-enzymatic and enzymatic detoxification methods in the different cell compartments would be required.

Complementing enzyme activity assays, metabolic flux analysis, histochemical activity staining, and redox status monitoring techniques provide a holistic view of metabolic capacities and regulatory mechanisms. Metabolic flux analysis links enzyme activity profiles with flux distributions in metabolic networks, employing isotope-based methods and mathematical models to predict metabolic responses to genetic and environmental changes [36,37,38]. Different methods allowing monitoring the cellular redox status in planta have been recently developed based on redox-sensitive fluorescent proteins, such as roGFP or rxYFP, for the analysis of H_2_O_2_, NAD(P)H, and glutathione redox status in different cell compartments [39]. In addition, a 2-Cys PRX biosensor allows the determination of its oxidation levels in the chloroplast, associated with photosynthesis performance [40]. However, although there has been substantial improvement in understanding how primed plants maintain redox homeostasis, so far only a few reports deal with the changes in redox regulation associated with plant-beneficial microbe interaction. For this, in addition to biochemical assays, genetic and molecular biology techniques, such as mutant analysis, gene expression analysis, and protein interaction studies, are essential. Mutant analysis elucidates the functional roles of genes involved in ROS metabolism and detoxification pathways, complemented by RNA sequencing (RNA-seq) to measure gene expression under oxidative stress [41,42]. Protein interaction studies, employing co-immunoprecipitation (Co-IP) and yeast two-hybrid (Y2H) assays, elucidate intricate networks of antioxidant enzymes vital for cellular defense mechanisms. Additionally, proteomics techniques, such as mass spectrometry (MS) and redox proteomics, provide comprehensive insights into ROS-responsive proteins, detailing their dynamics and modifications during stress conditions [43,44]. Advanced imaging methods like confocal microscopy and live-cell imaging play an important role in visualizing ROS production and distribution within plant cells, linking these dynamics to physiological outcomes under oxidative stress. This integrated approach establishes a robust framework for studying ROS and antioxidant responses in plants. It not only ensures precise experimental outcomes but also enhances our understanding of plants’ adaptive strategies against oxidative stress. This contributes significantly to advancements in the knowledge on plant stress physiology and resilience mechanisms.

Accordingly, Ameztoy et al. [45], based on redox proteomics and the analysis of mutants lacking different components of chloroplast redox detoxification, 2-Cys PRX, and redox regulation, NTRC, have identified thiol redox proteome changes affecting photosynthesis as a component of plant responses to fungal volatiles emitted by *Alternaria alternata*. On one hand, fungal volatile organic compounds (VOCs) failed to increase photosynthetic capacity in *ntrc* plants. On the other hand, the analysis of the redox proteome showed that whereas VOCs promote the reduction of the thiol redox proteome in wildtype plants, the effect in *ntrc* plants was the opposite, promoting protein oxidation. This effect was reverted when *ntrc* plants had reduced levels of 2-Cys PRXs, in the *ntrc-Δ2cp* mutant plants, showing that NTRC-dependent modification of the status of the redox proteome is key for plant responses to VOCs. Furthermore, the analysis of a mutant devoid of one of the plastidial isoforms of fructose-1,6-bisphosphatase, *cfbp1*, that is subjected to redox regulation, showed that fungal VOCs regulate photosynthetic plant responses through chloroplast-to-nucleus retrograde signaling [46]. In particular, *cfbp1* plants showed downregulation of plastidial Trxs and enzymes related to the methyl erythritol (MEP) pathway and photosynthesis and failed to show any growth promotion by VOCs.

## 3. Impact of Plant-Beneficial Microbe Interaction on Biological Processes Involving ROS and Redox Regulation

### 3.1. Growth and Development Under Normal and Abiotic Stress Conditions

ROS play a crucial dual role in plant growth and development. On one hand, ROS function as essential signaling molecules regulating key biological processes; on the other hand, as mentioned before, their overaccumulation can cause oxidative stress and cellular damage. Therefore, maintaining a delicate balance between ROS production and scavenging is fundamental for normal plant development. During plant growth, ROS are involved in multiple developmental stages, including seed germination, root elongation, shoot growth, and the differentiation of various tissues. For instance, H_2_O_2_, one of the primary forms of ROS, is known to participate in cell wall loosening during root hair formation and in the promotion of cell expansion. Furthermore, O_2_^−^ is crucial for controlling the rate of cell division in meristematic tissues. These ROS-mediated processes ensure proper organogenesis and are tightly regulated by both ROS-producing enzymes, such as NADPH oxidases, and antioxidant systems, such as SOD, CAT, and peroxidases (POX).

**Figure 2:**
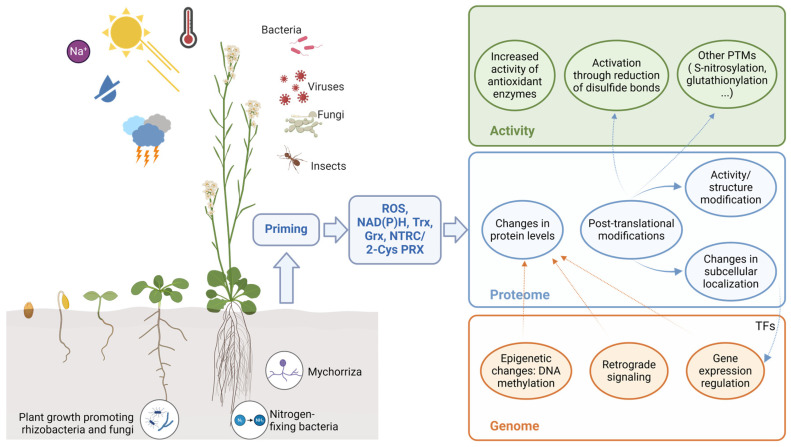
Effect of Plant-Growth-Promoting Rhizobacteria (PGPRs), Nitrogen-fixing bacteria, and Mychorriza on plant growth and resistance to abiotic and biotic stresses. The inoculation of the plant with microbes, which can be attracted by root exudates, alters nutrient availability, thus promoting plant growth and development. In addition, the microbes generate a primed state in the plants leading to enhanced resistance towards biotic and abiotic stresses. This effect is dependent on the control of ROS levels, through activation of enzymatic and non-enzymatic antioxidant mechanisms, and also on redox regulation mediated by Trxs, NTRC, and 2-Cys PRX. At the genome level, post-transcriptional modification of Cys alters subcellular localization of some TFs and results in the regulation of gene expression. PTMs of Cys residues, mediated directly or indirectly by ROS, also underlie operational retrograde signaling, allowing the adjustment of protein levels. Epigenetic modifications, such as DNA methylation, are emerging as key components in plant response to biotic stress and transgenerational priming, underlying the biocontrol activity of some microbes. At the proteome level, regulation of protein structure and/or activity through PTMs constitutes an additional layer of regulation. Dithiol–disulfide exchange of Cys residues results in the activation of key enzymes in photosynthesis. Some other PTMs, such as S-nitrosylation and glutathionylation, have been associated with gene transcription regulation and plant immunity. Figure was created in BioRender (https://BioRender.com/z93q802, accessed on 16 September 2024).

The inoculation of plants with plant-beneficial microbes typically results in an impact on the antioxidant machinery of plants. Through these interactions, beneficial microbes are expected to help plants manage oxidative stress and promote healthy development and contribute to coping with stress and support growth and development (Figure 2). This correlation was shown by activity profiling of antioxidant enzymes (POX, APX, CAT, SOD, GR, DHAR, and MDAR) [35] for the stimulation of growth of tomato by different *Pseudomonas* strains [42,47]. The effect of beneficial microbes on plant resistance to abiotic stress has been reviewed in several recent reports, with a special focus on the alleviation of salinity stress through the activation of antioxidant systems [48,49,50,51] and, therefore, will not be addressed in depth in this review. As an example, improvement of water use efficiency by *Bacillus licheniformis* FMCH001 [52] in maize, salt tolerance in quinoa by *Burkholderia phytofirmans* PsJN [53], and drought responses in tomato by *Pseudomonas fluorescens* G20-18 [42] correlated with specific antioxidant enzyme activity fingerprints compared to the corresponding mock controls. Also, at a whole microbiome level such correlations are evident. A correlation between the distinct difference in the activity profile of specific antioxidant enzymes of Abies plants of the same age but with different growth patterns and the relative abundances of specific taxa of the microbiome could be established [54].

In addition to the direct role of ROS in regulating plant development, ROS also interact with other signaling pathways that influence plant growth [55,56,57]. One such pathway is target of rapamycin (TOR) signaling, a central regulator of nutrient-dependent growth in plants. TOR signaling integrates sugar and light signals to drive plant growth, but its activity must be tightly controlled to prevent excessive growth at the cost of stress tolerance. Under nutrient-rich conditions, TOR promotes growth by stimulating anabolic processes, while during stress, TOR activity is inhibited, allowing the plant to allocate resources to survival mechanisms. The negative feedback loop involving the plant-specific protein FLZ8 is critical in moderating TOR activity [58]. FLZ8 reduces TOR signaling under unfavorable growth conditions, allowing for enhanced SnRK1 signaling, which is essential for stress responses. This regulatory balance ensures that plants can grow when resources are available but remain ready to activate stress responses during environmental challenges [59]. FLZ8 stabilizes SnRK1 signaling by promoting the interaction between SnRK1a1 and RAPTOR1B, a key component of TOR, thereby ensuring that TOR activity is kept in check during nutrient sufficiency [60]. This fine-tuned regulation prevents TOR hyperactivation and contributes to the plant’s ability to respond to stress. The dynamic balance between TOR and SnRK1, modulated by ROS and other signaling molecules, allows plants to prioritize growth or stress responses depending on environmental conditions. Recent studies have demonstrated that plant growth under stressful conditions, such as drought, salinity, or nutrient deficiency, is often accompanied by increased ROS generation [42,61,62]. However, plants primed with beneficial microbes exhibit enhanced stress tolerance due to their ability to quickly detoxify ROS [42,63]. This microbial modulation of ROS not only promotes plant growth under optimal conditions but also enhances growth resilience under environmental stresses. In addition to enzymatic antioxidant responses, beneficial microbes may induce the accumulation of non-enzymatic antioxidants such as ascorbate and glutathione, further contributing to the maintenance of redox homeostasis during plant development. The fungus *Acremonium alternatum* enhances salt stress tolerance by regulating host redox homeostasis in combination with phytohormone signaling [64].

In conclusion, the interaction between plants and beneficial microbes can improve plant growth and development by regulating ROS production and enhancing antioxidant responses. This symbiotic relationship provides an efficient system for plants to cope with oxidative stress, ensuring sustained growth under both normal and adverse environmental conditions. As a result, the incorporation of plant-beneficial microbes into agricultural practices presents a promising strategy for enhancing crop yield and resilience in the face of growing environmental challenges.

### 3.2. Disease Defense, Biocontrol, and Biopriming

Disease generated by different plant pathogens, such as bacteria, fungi, oomycetes, viruses, and herbivores, is one of the main causes of crop losses worldwide. Crop yield losses due to pathogens are estimated at USD 220 billion, a value further increased by the post-harvest losses caused by some pathogens [10], especially fungi, which affect all stages of crop production, accounting for 20–25% of total post-harvest losses [65]. Viruses and fungi are the main pathogens responsible for plant diseases, followed by pathogenic bacteria.

The development of a disease depends on the detection methods present in the plant that give rise to the different protection mechanisms but also on the evasion of detection by the pathogens that suppresses the plant defense response. In brief, recognition of the pathogens takes place primarily at the plasma membrane, where pathogen recognition receptors (PRRs) are able to detect some common pathogenic elements collectively named as pathogen-, microbe-, or herbivore-associated molecular patterns (PAMPs/MAMPs/HAMPs), such as bacterial flagellin or elongation factor Tu (EF-Tu) and fungal chitin, among others. In addition, in cell wall damage, producing glycans or an increase in the extracellular levels of ATP, so-called damage-associated molecular patterns (DAMPs) can be detected by PRRs, triggering a defense response. This response, known as PRR-mediated immunity (PMI), extracellular triggered immunity (ExTI), or surface-receptor-mediated immunity (SRMI), is characterized by a signaling cascade involving the activation of protein kinases. Evolution of pathogens allows the suppression of PMI by the production of effector molecules, leading to effector-triggered susceptibility (ETS). Plants in turn have evolved resistance genes coding for intracellular nucleotide-binding leucine-rich repeat receptors (NLRs), which can recognize effectors and promote a defense response denoted as NLR-mediated immunity (NMI), intracellular triggered immunity (InTI), or intracellular-receptor-mediated immunity (IRMI). This response requires in some cases the assistance of helper NLRs which activate immune responses by formation of calcium channels and activation of transcription factors. Although specific signaling components participate in PRR- and NLR-mediated immunity, recent studies suggest that there is an interdependency between them [66]. In any case, the plant–pathogen interaction determining susceptibility to or resistance against a particular pathogen of a crop is under constant co-evolution, with the development of new recognition receptors or resistance genes by the plants and new effectors that challenge plant immune systems by the pathogen.

However, the development of a disease depends not only on the plant and the pathogen itself but also on the environmental and culture conditions, with high-density crops facilitating the spread of pathogens. The environmental conditions affect plant–pathogen interaction in multiple ways. First, by altering pathogen dynamics, favoring its survival and replication and leading to the appearance of new more aggressive strains due to the increases in temperature associated with global warming. Second, by altering plant physiology and biochemistry, leading to enhanced susceptibility due to the suppression of salicylic acid production or increases in abscisic and jasmonic acid biosynthesis under high temperatures. Third, by altering the expression of hormone-responsive genes within the plant cell [10]. Besides temperature, two other factors associated with climate change affect pathogen infectivity, atmospheric carbon dioxide levels and water availability. The increase in atmospheric CO_2_ positively affects crop yield but, on the other hand, has been shown to increase wheat susceptibility towards *Fusarium graminarearum*, powdery mildew severity on cucurbits, and head blight and blotch on wheat. However, in other cases the severity of the disease was reduced in these conditions, so a general assumption on the effect of CO_2_ concentration on pathogen spread cannot be drawn. Higher temperatures increase the amount of water vapor in air after rain, promoting the infectivity of pathogens affecting aerial tissues, mainly fungi. On the contrary, the effect of soil moisture levels on diseases provoked by soil-inhabiting pathogens is diverse. In any case, the combined effect of these environmental factors could have altered the plant immunity mechanisms described before [67].

The generation of ROS associated with the interaction of plants with different types of pathogens, such as bacteria, fungi, and viruses, is essential for triggering signaling cascades that contribute to plant resistance. Already at the plant cell surface ROS are produced by a family of NADPH oxidases, known as respiratory burst oxidase homologs (RBOHs), activated upon pathogen recognition by PRRs, in a process that seems to be pathogen specific. In addition, apoplastic POXs contribute to the generation of ROS. In fact, the production of ROS by POXs seems to be required for the activation of NADPH oxidases and the consequent oxidative burst. Moreover, it has been suggested that H_2_O_2_ and superoxide anion produced by POXs and RBOHs, respectively, can specifically activate signaling pathways leading to plant resistance [68,69,70]. A second-wave production of ROS has been linked to the interaction with avirulent pathogens associated with NLR-mediated immunity, resulting in the hypersensitivity response, crosslinking of the membrane cell wall, deposition of callose, and stomatal closure. However, a reduced accumulation of H_2_O_2_ was observed in compatible interactions of wheat with *Septori tritici* [71], showing that ROS production is a key element in wheat defense. Although the inhibition of biotrophic pathogens by H_2_O_2_ is clear, its effects on necrotrophic pathogens are diverse [70]. While chloroplasts, mitochondria, and peroxisomes contribute to the generation of ROS, the chloroplast is the main organelle responsible for ROS production. Within the chloroplast, the effect of different bacterial effectors alters electron transport and ROS production resulting in pathogenesis, showing that plastidial production of ROS is essential for plant defense [23]. Furthermore, plastidial ROS generation alters redox homeostasis in the cytosol and mitochondria [72]. In addition to oxidative burst and hypersensitive response, apoplastic ROS produced by RBOHs also underlie systemic acquired resistance [73].

Oxidation of Cys residues due to pathogen-induced ROS has been described as related to plant immunity responses (Figure 2). This modification can alter protein structure/function, localization, and stability. As an example, the release of non-expressor of pathogenesis-related genes 1 (NPR1) monomers by reduction mediated by TRX *h* is essential for its translocation to the nucleus and the elicitation of transcriptional regulation [74], showing that an adequate shaping of the redox proteome is essential to plant immunity. In addition, the formation of NPR1 oligomers is favored by protein nitrosylation promoted by the pathogen. RBOHD activity, and the subsequent ROS production, is impaired by Cys nitrosylation [75]. ROS-induced oxidation is also necessary to activate MAPK cascades [76]. Finally, oxidation of HPCA, a receptor kinase involved in stomata closure under stress, is necessary for its activation by autophosphorylation [76,77]. In addition, since Trxs can revert the oxidation of Cys, not only of disulfide but also of sulfenic and nitrosothiols, a key role of these proteins in immune response was suggested, that is supported by experimental evidence [23,78]. Taking into account that the oxidation of proteins is dependent on the ROS levels, the maintenance of the ROS balance is essential for the plant response toward pathogens. Consequently, antioxidant enzymes (CAT, APX, POX, PRX) and non-enzymatic antioxidants (ascorbate and glutathione) are suggested to play a key role in the immune response [23,79,80]. Interestingly, some of the antioxidant enzymes, cytosolic APXs and PRXs, have been reported to be regulated by oxidative modification [23].

Priming plants for a quicker and better response to pathogens constitutes a promising and sustainable alternative to the use of pesticides, overcoming, in addition, the development of pathogen resistance. Besides natural active compounds, such as vitamins, hormones, or hormone antagonists, the use of beneficial fungi and bacteria as biostimulants, promoting plant growth, and biocontrol agents, promoting plant resistance, has gained attention in the last decade [15,81]. Both microbes naturally occurring in the rhizosphere, endophytes, or plant-growth-promoting rhizobacteria (PGPRs) and microbes incorporated from external sources can be used as biocontrol agents (BCAs); this microbial consortium allows a more effective and durable pathogen control [82,83]. Besides antibiosis, antagonism, competence, and the induction of acquired and induced systemic resistance (SAR and ISR), increased ROS detoxification and maintenance of redox homeostasis are different strategies used by microbial BCAs, including bacteria (such as *Bacillus subtilis*, *Agrobacterium* sp., and *Pseudomonas fluorescens*) [84] and fungi (such as *Trichoderma*, *Aspergillus*, and *Penicillum*), to limit disease produced by plant pathogens. In fact, the ISR pathway activated by BCAs is associated with the enhanced activity of POXs, as important defense proteins, along with diminished ROS accumulation [85].

PGPRs are usually used as BCAs since, in addition to their ability to promote plant growth, they can enhance plant resistance towards phytopathogens (Figure 2). Bacterial strains belonging to the genera *Bacillus* and *Pseudomonas* have been shown to increase antioxidant activity within the plant, thus regulating ROS levels, resulting in plant resistance. In tomato seeds, treatment with *Pseudomonas fluorescens* VSMKU3054 promoted resistance against *Ralstonia solanacearum* associated with an increased POX activity when primed seeds were challenged with the phytopathogen [86]. In chili plants, volatile organic compounds (VOCs) produced by a consortium of *Pseudomonas fluorescens* PDS1 and *Bacillus subtilis* KA9 resulted in an enhanced protection towards *R. solanacearum* associated to the overexpression of antioxidant enzymes and increased SOD and POX activity [87]. Culture filtrates of *Bacillus subtilis* also resulted in an increase in POX activity and increased resistance towards *Xhantomonas campestris* and *Pseudomonas syringae* pv. tomato in tomato plants [88]. The induction of antioxidant enzymes produces in turn callose deposition and cell wall thickening, as well as phenolic compound accumulation, which fosters plant resistance to phytopathogens.

Besides protecting plants against bacterial phytopathogens, numerous bacteria inhibit growth of phytopathogenic fungi though a mechanism involving an increased activity of antioxidant enzymes for the maintenance of an adequate redox balance. In tomato, plant mortality caused by the collar rot fungus *Sclerotium rolfsii* was reduced due to incubation with a consortium of *Bacillus subtilis* and *Pseudomonas fluorescens*, which allowed ROS scavenging through increased ascorbate content and augmented SOD, APX, POX, GRX, and CAT activities [89]. Similarly, different *Bacillus* strains isolated from a healthy rice plant within a false-smut-affected zone, caused by *Ustilaginoidea virens*, were shown to increase SOD and CAT activity [90], and seed coating with *Bacillus aryabhattai* Z-48 in tomato increased POX activity and suppressed *Fusarium oxysporum* f. sp. *lycopersici* [91]. Finally, *Bacillus* has also been shown to promote fruit resistance to fungal pathogens through the increase in antioxidant capacity [90]. This mechanism of induced resistance is also observed in biocontrol with different *Streptomyces strains:* An increased expression and activity of SOD, POX, and CAT due to priming with a consortium of two *Streptomyces araujoniae* strains (TN11 and TN19) resulted in reduced H_2_O_2_ levels and reduced lipid peroxidation in chickpea challenged with *Fusarium oxysporum* f. sp. ciceris [92]; an increased CAT activity was also reported due to *Streptomyces consortia* in chickpea plants challenged with *Fusarium* wilt [93]; POX activity was enhanced as well in *Fusarium*-wilt-infected cucumber plants when primed by *Streptomyces bikiniensis* HD-087 [94]. Finally, *Pseudomonas protegens* ML15 protected tomato from post-harvest gray mold caused by *Botrytis cinerea* BC21, through reduction of H_2_O_2_ levels and increased ascorbic acid and antioxidant activities [95].

Control of ROS levels is also a component of fungi used as biocontrol agents for fungal and microbial phytopathogens. Within fungi, *Trichoderma* is considered a very effective biocontrol agent suppressing plant diseases. In tomato, avirulent *Rhizoctonia* protects the plant from infection produced by a broad range of fungi, acting as a biocontrol agent against *Rhizoctonia solani,* preventing accumulation of H_2_O_2_, and increasing enzymatic antioxidant activities, including CAT, POX, and SOD, thus interfering with the pathogenesis strategy of the virulent multinucleate necrotrophic *R. solani* [96]. In rice a consortium of *Trichoderma harzianum* and the bacterium *Pseudomonas fluorescens* reduced H_2_O_2_ levels, by means of increasing POX and SOD activity, leading to increased defense against *Rhizoctonia solani* Kühn [97]. Similarly, *T. viride* together with the bacterium *Bacillus subtilis* increased POX activity and reduced disease severity of *Xhantomona campestris* and *Pseudomonas syringae* pv. tomato in tomato plants [88].

In *Arabidopsis* it has been shown that commensal bacteria isolated from healthy plants trigger diverse patterns of ROS production which activates a negative feedback loop between Arabidopsis ROS and the bacterial type II secretion system, thus taming a potential detrimental leaf commensal into a microbe beneficial to the host [98]. Seed inoculation with entomopathogenic fungi causes plant-mediated effects against arthropod herbivores. Antioxidant enzyme regulation patterns in fungus-inoculated wheat reflected resistance and tolerance towards an arthropod insect herbivore [99], supporting the relevance of such pathosystems.

Altogether, different reports describe the effect of BCAs both of fungal and bacterial natures on the plant antioxidant machinery, leading to a decreased accumulation of H_2_O_2_ that interferes with the pathogen virulence strategy and promotes induced systemic resistance in the plant. It is noteworthy to mention that most of the activity assays here reviewed were performed based in crude extracts, and whereas some of the enzymatic antioxidants such as CAT have a unique localization, other enzymatic activities, such as APXs and SODs, are localized to different cell compartments. Therefore, a more in-depth analysis of the subcellular changes related to these enzymes could help to draw a more complete image of what is happening within the plant, especially in relation to the effect of BCAs on protection against bacterial pathogens, which alter chloroplast ROS levels through the activity of their effectors, since the adequate homeostasis of ROS in the chloroplast is of paramount importance for photosynthesis. In gel activity assays for both SOD [28,100] and APX [101] are available which allow one to distinguish the increases in the different isoforms of the enzymes. Additionally, very little information is available on the impact of BCAs on chloroplastic redox regulation, an aspect that deserves further research.

### 3.3. Symbiosis

The interaction between plants and beneficial symbiotic associations plays a crucial role in enhancing plant resilience to various stresses through complex biological processes. These microbes, including mycorrhizal fungi like *Glomus* spp., *Piriformospora indica*, and *Rhizobium* bacteria, assist plants by fixing nitrogen and facilitating nutrient uptake [102,103,104,105,106]. The establishment of the symbiotic interaction is closely linked with the regulation of ROS. During the legume–Rhizobium interaction, ROS play essential roles in root hair curling, infection thread formation, and nodule development [103,107]. For example, ROS production is necessary for the rigidity of infection threads, which facilitates the progression of rhizobia into the plant cortex [108]. In *Sesbania rostrata*, ROS accumulation leads to localized cell death and the formation of infection pockets, crucial for nodule initiation [109]. Similarly, mycorrhizal associations reduce ROS levels in plant tissues, thereby mitigating oxidative damage and enhancing stress tolerance. Studies have shown that arbuscular mycorrhizal fungi (AMF) colonization decreases H_2_O_2_ accumulation and lipid peroxidation under stress, thereby improving plant growth under adverse environmental conditions [110,111,112]. Mycorrhizal fungi have been shown to reduce ROS levels in roots under stress conditions, thereby enhancing root system architecture and promoting better nutrient uptake [113,114].

In addition to facilitating nutrient acquisition, mycorrhizal fungi play a crucial role in modulating antioxidant responses and redox regulation within the plant. The symbiosis between AMF and plant roots enhances the plant’s ability to mitigate oxidative stress through the upregulation of genes encoding antioxidant enzymes like SOD, CAT, and POX, thereby preventing cellular damage under stress conditions [115]. Mycorrhizal fungi not only reduce ROS accumulation but also maintain redox homeostasis by modulating the activity of these antioxidant systems. This balance between ROS production and scavenging is crucial for maintaining the integrity of cellular structures and supporting normal physiological processes during stress conditions such as drought, salinity, and nutrient deficiency [114,116]. Furthermore, studies have shown that AM symbiosis promotes the synthesis of non-enzymatic antioxidants such as ascorbate and glutathione, which are integral components of the plant’s defense against oxidative stress [117,118]. The interaction of AM fungi with plants triggers a series of molecular events that optimize both nutrient uptake and stress resilience. For instance, AM fungi enhance water uptake by extending the hyphal network into soil micropores, inaccessible to plant roots, helping alleviate drought stress. At the same time, the symbiosis improves nutrient acquisition (especially phosphorus and nitrogen), which indirectly bolsters the plant’s antioxidant capacity. This coordinated effort between nutrient uptake and redox regulation highlights the role of AM fungi in strengthening the plant’s adaptive response to environmental challenges [114].

Likewise, in the case of rhizobial symbiosis, *Rhizobium* bacteria form nodules in legume roots, where nitrogen fixation takes place. This process is tightly controlled by redox regulation, as the nitrogen-fixing enzyme nitrogenase is sensitive to oxidative stress. In the nodules, ROS and RNS, such as O_2_^−^, H_2_O_2_, and NO, must be carefully managed to balance signaling functions with protection against oxidative damage. Rhizobial bacteroids, the differentiated bacterial forms inside the nodules, are equipped with antioxidant enzymes, such as SOD, CAT, Prx, and Grx, which detoxify ROS/RNS and maintain redox homeostasis during nitrogen fixation [119,120]. Trx and Grx play key roles in the redox regulation of retrograde signaling and gene transcription during symbiosis, through the regulation of redox-sensitive transcription factors, maintaining a cellular redox state through post-translational modifications of Cys residues. In nodules, Trx and Grx are involved in disulfide exchange reactions, modulating the activity of proteins necessary for nodule formation and nitrogen fixation [119,121]. For instance, Trx proteins reduce the oxidative modifications of transcription factors and other proteins, ensuring proper nodule development and functioning. Grxs are responsible for protein deglutathionylation, a process critical for maintaining protein function and structure under oxidative stress. Moreover, Trx and Grx systems participate in retrograde signaling, where the redox status in nodules and bacteroids sends signals back to the nucleus, influencing the expression of genes involved in nodule development and stress responses. This retrograde regulation ensures that nodule formation, nitrogen fixation, and antioxidant defense systems are finely tuned, allowing the plant to optimize its symbiotic interaction with rhizobia, even under challenging environmental conditions. Furthermore, rhizobia activate genes such as CAT in response to oxidative stress within nodules, helping protect both the bacteria and plant from oxidative damage, thus sustaining efficient nitrogen fixation [119].

In symbiotic interactions, maintaining a balance between the production and scavenging of ROS is crucial for cellular redox homeostasis. Symbiosis induces the expression of genes that regulate antioxidant responses, such as rhizobium-induced peroxidases like *Rip1* and *Srprx1* in *Medicago truncatula*, which are upregulated during symbiosis [122]. Rhizobia have evolved specific adaptations to cope with the oxidative environments within nodules. For example, *Sinorhizobium meliloti* is known to overexpress CAT, thereby protecting these bacteria from oxidative damage [123]. This adaptation plays an important role in preserving the symbiotic relationship by mitigating oxidative stress, which is crucial because the elevated levels of ROS within nodules pose a challenge to nitrogenase. This highlights the necessity of such bacterial adaptations to sustain efficient nitrogen fixation despite oxidative stress conditions. *Piriformospora indica* activates the glutathione-ascorbate cycle and increases the levels of SOD, POD, and CAT, thus increasing barley resistance to fungal diseases [124].

Furthermore, ROS signaling pathways activated during symbiotic interactions induce the expression of defense-related genes and adaptive responses in plants, such as pathways involving OXI1 kinase in response to oxidative stress and defense against pathogens [105]. The modulation of ROS metabolism by beneficial symbiosis not only contributes to improved plant fitness, resilience, and yield but also enhances symbiotic relationships. Therefore, understanding the interplay of antioxidant responses and redox regulation in plant–microbe symbiotic interactions offers promising ways for enhancing plant productivity and sustainability.

## 4. Perspectives and Open Questions

As described before, most of the studies performed on the effect of beneficial microbes on plant growth and resistance to biotic stress are focused on the activation of different antioxidant mechanisms. However, many other processes are emerging in the literature as being regulated by beneficial microbes, contributing to plant resilience to stress (Figure 2).

One of the mechanisms allowing acclimation to stress situations is the adjustment of gene expression. ROS-regulated gene expression and its contribution to priming have been extensively studied and reviewed, so describing it in depth is out of the scope of this manuscript [15,22,125]. The contribution of Trx to gene expression regulation under stress conditions through redox modifications of transcription factors (TFs) is well known. Post-translational modification of Cys residues results in the translocation of some TFs from the cytoplasm to the nucleus under stress conditions. In addition, redox-dependent changes in the oligomerization status of TFs are responsible for the regulation of their activity. Interestingly, Trx *o*1 and *h* and NTR have also been shown to localize to the nucleus [22,126]. An aspect that merits special attention is retrograde signaling under adverse conditions, especially in relation to biotic stress and plant-beneficial microbe interaction. This process, essential for the coordinated regulation of nuclear and organelle gene expression in photosynthetic organisms under adverse conditions, the so-called operational control, is mediated, among other metabolites, by ROS, either directly or through post-translational modification of Cys residues [22]. Retrograde signaling has been an important subject of analysis in relation to plant and cyanobacteria acclimation to abiotic stress since the beginning of this century, with a special focus on chloroplasts, due to their contribution to ROS production and the photo-oxidative damage associated with abiotic stress. Several recent manuscripts report the importance of Cys modifications in operational retrograde signaling. The repair of PSII through the activity of ATP-dependent zinc metalloproteases, FtsH1 and FtsH2, depends in *Chlamydomonas reinhardtii* on the formation of an active oligomer through the reduction of disulfide bridges, suggesting a possible regulation by Trxs not only in microalgae but also in plants [127]. Import of chloroplast proteins during organelle biogenesis, mediated by the chaperone cpHSP70, via interaction with the GUN1-cpHSP70 complex, is regulated by the NTRC/2-Cys PRX system [20]. Additionally, the reductive activation of Plastid Redox Insensitive2 (PRIN2) dimers, mediated by plastidial *f*1 and *z* Trxs, is necessary for the activation of the plastid-encoded RNA polymerase [128]. *f* and *m* Trxs and NTRC also regulate the activity of Mg-chelatase I and H subunits (CHLI and CHLH) and other upstream enzymes in chlorophyll biosynthesis [21,129,130], leading to the accumulation of Mg-protoporphyrin IX, thus triggering retrograde signaling. The full significance of other modifications of Cys residues, such as oxidation to sulfonic acid or glutathionylated forms in retrograde signaling, remains to be fully determined. Recent reports show that glutathionylation of 3′-phosphoadenosine 5′-phosphate (PAP) phosphatase (SAL1), as well as intramolecular disulfide formation, suppresses its phosphatase activity, resulting in the accumulation of 3′-phosphoadenosine 5′-phosphate, which acts as a signal from the chloroplast to the nucleus, pointing out the importance of other PTMs in retrograde signaling. Additionally, SAL1 activity is altered in *ntrc* mutant plants, suggesting a redox regulatory role for the Trx system [131]. However, the contribution of retrograde signaling in the adjustment of nuclear and organellar gene expression in relation to the response to biotic stress and the effect of BCAs in the modulation of operational control remains, to our knowledge, to be determined.

Besides regulation of gene expression, epigenetic modifications in DNA and histone proteins are recently emerging as key components in plant response to biotic stress and transgenerational priming, and both ROS-induced DNA hypomethylation and histone modifications have been reported [132]. First, silencing of pathogen genes through epigenetic modifications is part of the plant defense mechanism. Second, DNA methylation and histone modifications allow the plants to fight stresses through reprogramming gene expression, generating a stress memory that results in increased survival under stress circumstances in the next generations. In Arabidopsis, inheritable methylation of two adjacent NLR genes generates resistance against *Plasmodiophora brassicae* [133] and transgenerational epigenetic variations also underlie quantitative resistance towards clubroot infection [134]. In wheat, a histone acetyltransferase TaHAG1 contributes to the resistance against powdery mildew by the regulation of transcription of the transducer protein Phytoalexin Deficient 4 (PAD4) [135]. In Arabidopsis, increased methylation of the promoter region of the WRKY53 transcription factor is related to its redox-dependent induction in response to infection by *Pseudomonas syringae* pv Maculicola [136]. Epigenetic modifications also underlie the biocontrol activity of some microbes. Gkizi et al. [137] reported the contribution of histone acetylation to the biocontrol activity of the PGPR *Paenibacillus alvei* K165 against *Verticillum dahliae,* through the expression of immune genes and lignin deposition, that was inherited in the offspring. This is an aspect that deserves further attention to fully understand the significance of epigenetic modification in biocontrol and biopriming.

An important level of regulation in the response of plants to stress is redox regulation of key proteins, especially of chloroplast proteins affecting photosynthesis and, therefore, plant performance. Although the NTRC/2-Cys Prx system plays a fundamental role in regulating the redox status of the chloroplast, which is essential for the photosynthetic activity of the plant, and, as mentioned before, plant inoculation with microorganisms produces an increase in antioxidant activities related to higher stress tolerance of treated plants, not many studies to date have addressed the effect of growth-promoting microorganisms on the redox state of thiol antioxidant and other redox-regulated enzymes. The results obtained by Ameztoy et al. [45,46] show that redox regulation mediated by NTRC/2-Cys PRX is key to the effect of the VOCs emitted by the fungus *Alternaria alternata*. However, very scarce information on the contribution of redox regulation to the effect of beneficial microbes on plant growth and resistance to biotic stress is available and detailed analysis, through mutant analysis, redox proteomics, and alkylation assays for the analysis of the redox status of individual proteins, is needed to fully understand the importance of redox regulation.

To sum up, the interaction between plants and beneficial microbes can improve plant growth and resilience through the maintenance of ROS homeostasis and redox regulation (Figure 2), an aspect that deserves further research. Efforts must be undertaken to analyze the effect of simultaneous stresses on plant performance, to mimic the actual conditions plants face under the current environmental conditions. Evaluation of the effect of beneficial microbes under those conditions would help to establish sustainable practices for the mitigation of the effects of climate change on plant growth and productivity.

## 5. Conclusions

The use of beneficial microbes to promote plant growth and resilience towards biotic and abiotic stresses has been established in the last two decades. This review highlights current knowledge about antioxidant mechanisms driven by microbes, essential for maintaining ROS balance and improving plant stress tolerance. Processes such as the activation of enzymatic antioxidants and non-enzymatic systems are discussed in detail. Additionally, we have emphasized the critical role of redox regulation in activating immune responses through transcriptional regulation and post-translational modifications. These areas are key to understanding the biocontrol effects of microbes. However, redox regulation within plant–PGPR interactions, especially its impact on thiol-based modifications, protein changes, and retrograde signaling, remains underexplored. While evidence suggests redox regulation plays a role in photosynthesis, stress adaptation, and immune priming, more systematic studies using advanced tools like redox proteomics, live-cell imaging, and genetic approaches are needed.

Additionally, research will be necessary to address security issues, namely the effect on non-target native soil communities, especially in relation to long-term applications of non-native microorganisms. Though an effect of biologically based inocula on the resident soil microbial community has been described in several studies, how this affects ecosystem functionality remains to be determined. The use of different multi-omics approaches is a promising tool for this goal [138]. The research on the presence of antibiotic resistance genes in some PGPRs and the potential transference of these genes to other soil microbes, another limitation of the use of beneficial microbes in agriculture, is still in its infancy [139].

Future research should focus on integrating redox regulation with antioxidant responses to build a more complete understanding of plant–microbe interactions. This integrated approach can guide the development of sustainable agricultural practices, using PGPRs to improve crop resilience and address global challenges such as climate change and food security.
